# Crystal structure of the translation recovery factor Trf from *Sulfolobus solfataricus*

**DOI:** 10.1002/2211-5463.12772

**Published:** 2019-12-18

**Authors:** Marco Kaiser, Jan Philip Wurm, Birgit Märtens, Udo Bläsi, Denys Pogoryelov, Jens Wöhnert

**Affiliations:** ^1^ Institute of Molecular Biosciences and Center for Biomolecular Magnetic Resonance (BMRZ) Goethe‐University Frankfurt Germany; ^2^ Department of Microbiology, Immunobiology and Genetics Max F. Perutz Laboratories Center of Molecular Biology University of Vienna Austria; ^3^ Institute of Biochemistry Goethe‐University Frankfurt Germany; ^4^ Present address: Institute of Biophysics and Physical Biochemistry University of Regensburg Germany

**Keywords:** DUF35, ribosome, *Sulfolobus solfataricus*, translation initiation, translation recovery factor Trf

## Abstract

During translation initiation, the heterotrimeric archaeal translation initiation factor 2 (aIF2) recruits the initiator tRNA_i_ to the small ribosomal subunit. In the stationary growth phase and/or during nutrient stress, *Sulfolobus solfataricus* aIF2 has a second function: It protects leaderless mRNAs against degradation by binding to their 5′‐ends. The *S. solfataricus* protein Sso2509 is a translation recovery factor (Trf) that interacts with aIF2 and is responsible for the release of aIF2 from bound mRNAs, thereby enabling translation re‐initiation. It is a member of the domain of unknown function 35 (DUF35) protein family and is conserved in Sulfolobales as well as in other archaea. Here, we present the X‐ray structure of *S. solfataricus* Trf solved to a resolution of 1.65 Å. Trf is composed of an N‐terminal rubredoxin‐like domain containing a bound zinc ion and a C‐terminal oligosaccharide/oligonucleotide binding fold domain. The Trf structure reveals putative mRNA binding sites in both domains. Surprisingly, the Trf protein is structurally but not sequentially very similar to proteins linked to acyl‐CoA utilization—for example, the Sso2064 protein from *S. solfataricus*—as well as to scaffold proteins found in the acetoacetyl‐CoA thiolase/high‐mobility group‐CoA synthase complex of the archaeon *Methanothermococcus thermolithotrophicus* and in a steroid side‐chain‐cleaving aldolase complex from the bacterium *Thermomonospora curvata*. This suggests that members of the DUF35 protein family are able to act as scaffolding and binding proteins in a wide variety of biological processes.

## Graphical abstract

The X‐ray structure of the archaeal translation recovery factor (Trf) reveals a two‐domain protein with a putative mRNA‐binding cleft. The N‐terminal domain resembles the rubredoxin fold, whereas the C‐terminal domain has an oligosaccharide/oligonucleotide binding fold. Trf is structurally similar to other proteins of the domain of unknown function 35 family that have functions not related to RNA binding.

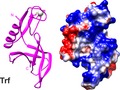

**Abbreviations**
aIF2: archaeal initiation factor 2
DUF: domain of unknown function
EMSA: electrophoretic mobility shift assay
OB‐fold: oligosaccharide/oligonucleotide binding fold
PDB: Protein Data Bank
Trf: translation recovery factor

*Sulfolobus solfataricus* is a thermoacidophilic archaeon originally isolated from hot volcanic springs. It grows optimally at 350 K and in acidic environments with a pH between 2 and 4 1. Fundamental features of translation initiation are conserved between archaea such as *S. solfataricus* and eukaryotes 2. The heterotrimeric translation initiation factor IF2 (α, β, and γ) is required for binding of the methionine‐loaded initiator tRNA (tRNA_i_) to the small ribosomal subunit as well as the proper positioning of tRNA_i_ in the ribosomal P‐site in archaea and in eukaryotes. However, crenarchaeal archaeal initiation factor 2 (aIF2) lacks the tRNA_i_ shuttle function of eukaryotic initiation factor 2 3, 4. Interestingly, in *S. solfataricus* the aIF2γ subunit has an additional function during the stationary growth phase and/or during nutrient starvation. Under these conditions, it protects mRNAs against exonuclease‐mediated 5′–3′ decay via binding to their triphosphorylated 5′‐ends 5, 6. To reinitiate translation after nutrient replenishment, aIF2γ must be displaced from mRNAs. The small protein translation recovery factor (Trf) was identified as the factor responsible for releasing aIF2γ from bound mRNAs, thereby enabling re‐initiation of translation after relief of nutrient stress 5.

A sequence alignment of Trf homologs from different *Sulfolobus* species revealed two conserved regions predicted to be similar to an N‐terminal rubredoxin‐like and a C‐terminal oligosaccharide/oligonucleotide binding fold (OB‐fold) domain 5. Here, we report the crystal structure of the 124‐amino acid (aa) *S. solfataricus* Trf protein (Sso2509) at a resolution of 1.65 Å. Interestingly, the structure but not the sequence of Trf is similar to other proteins of the domain of unknown function 35 (DUF35) family [Protein Data Bank (pdb): pdb http://www.rcsb.org/pdb/search/structidSearch.do?structureId=3irb, http://www.rcsb.org/pdb/search/structidSearch.do?structureId=6et9 chain E, http://www.rcsb.org/pdb/search/structidSearch.do?structureId=6ok1 chain B, http://www.rcsb.org/pdb/search/structidSearch.do?structureId=5m3k chain B] that have been described to bind directly or indirectly to acyl‐CoA 7, 8 or act as scaffolding components of acyl‐CoA‐dependent enzyme complexes 8, 9 as well as a CoA‐independent bacterial acylase 10. This suggests a general role of DUF35 family proteins as scaffolding or binding proteins in a variety of biological processes.

## Materials and methods

A codon‐optimized gene for production of Trf in *Escherichia coli* was obtained commercially from GenScript in a pUC57 plasmid vector. The gene encoded the full‐length native Trf protein sequence from *S. solfataricus*. The coding region was subcloned into a pET11a expression plasmid using the NdeI and BamHI restriction sites.

*Escherichia coli* BL21 (DE3) cells were transformed with this plasmid, and cells were grown at 37 °C in Luria broth medium supplemented with 100 mg·mL^−1^ ampicillin until an optical density (OD_600_) of 0.8 was reached. Trf synthesis was induced by adding 1 mm isopropyl β‐d‐1‐thio‐galactopyranoside at 20 °C. Sixteen hours after induction, the cells from a 1 L culture were harvested by centrifugation at 5000 ***g*** (10 min, 4 °C) and disrupted by sonication in 20 mL lysis buffer (50 mm BisTris pH 5.8, 200 mm NaCl, 10 mm ß‐mercaptoethanol, 200 mm imidazole) on ice. After cell lysis, a heat denaturation step was performed at 65 °C for 15 min. Subsequently, the solution was centrifuged at 7000 ***g*** (20 min, 4 °C). The supernatant containing Trf was diluted with dilution buffer (50 mm BisTris pH 5.8, 10 mm ß‐mercaptoethanol, 200 mm imidazole) to 150 mL and loaded onto a SP‐Sepharose column (GE Healthcare, Solingen, Germany, HiPrepTM SP FF 16/60). Trf was eluted with a buffer containing 1 m NaCl in a linear gradient. Fractions containing Trf were pooled, concentrated to 4 mL, and applied to a gel filtration column (Superdex 200 HR 26/60; GE Healthcare) pre‐equilibrated with gel filtration buffer (50 mm BisTris pH 6.5, 50 mm NaCl, 5 mm ß‐mercapthoethanol, 200 mm imidazole). Fractions containing Trf were pooled and concentrated to a final protein concentration of 175 μm. Protein purity was assessed by SDS/PAGE. MALDI mass spectrometry yielded a molecular weight of 14.78 kDa (calculated 14.78 kDa).

Crystallization of Trf was achieved by slowly removing imidazole from the buffer during a two‐step dialysis of a gel filtration sample (4 mL/175 μm) of Trf against 1 L of an imidazole‐free dialysis buffer (50 mm BisTris pH 6.5, 50 mm NaCl, 5 mm ß‐mercapthoethanol) at 4 °C in a dialysis volume of 500 mL. After 24 h, the buffer was exchanged, and dialysis was continued for additional 72 h. After 4 days of dialysis, crystals appeared in the dialysis chamber (Slide‐A‐Lyzer™, Thermo Fisher, Waltham, MA, USA, 3.5 kDa molecular weight cutoff). A microscopic analysis revealed rod‐like crystals being UV‐active. These crystals were used for structure determination. The crystals were cryoprotected by adding 33% PEG‐400 to the crystallization solution.

X‐ray diffraction data were collected on the beamline station PXIII of the Swiss Light Source (Paul Scherrer Institut, Villigen, Switzerland). All diffraction data were obtained from a single crystal and processed with the xds software package 11. The positions of anomalous scatterers were determined using SHELXD 12. The initial phases were obtained by shelxe 13, 14, and the initial model was built automatically by resolve 15. The initial model was extended by iterative rounds of model building with coot 16 into the 2F_o_–F_c_ electron density map and refined using the phenix software package 17. The Ramachandran plots of the final structure showed no outlying residues, as assessed by model validation with the program molprobity 18. The graphical representations were generated using chimera 19. Proteins that are structurally similar to Trf were identified using the DALI server 20 and PDBeFOLD 21. Cα RMSDs and *Z*‐scores were calculated using the pairwise structure comparison option available at the DALI server.

Fluorescence anisotropy‐based RNA binding assays were performed at 25 °C using a Fluorolog 3 spectrometer (Horiba, Bensheim, Germany). Excitation and emission wavelengths were set to 492 and 521 nm. 5′‐fluorescein‐labeled RNAs were obtained commercially (Dharmacon, Lafayette, CO, USA) and deprotected according to the instructions of the manufacturer. Experiments were performed in a buffer containing 50 mm BisTris, pH 6.5, and 100 mm NaCl. A NaCl concentration of 100 mm in the buffer was necessary to prevent precipitation of the protein. The concentrations of the 5′‐fluorescein‐labeled RNAs were 100 nm. Trf was added stepwise until saturation was reached. Titration experiments were performed in triplicate. Each data point represents the average value with error bars indicating the standard deviation.

Analytical gel filtration in a buffer containing 50 mm BisTris, pH 6.5, and 150 mm NaCl was performed as described previously 22. A protein concentration of 50 μm was used. For molecular weight comparisons, we used the proteins *Ph*S11 and *Ph*Fap7 as well as the highly stable PhFap7/PhS11 complex with molecular weights of 14.74, 20.18, and 34.92 kDa, respectively 18.

## Results and Discussion

**Figure 1:**
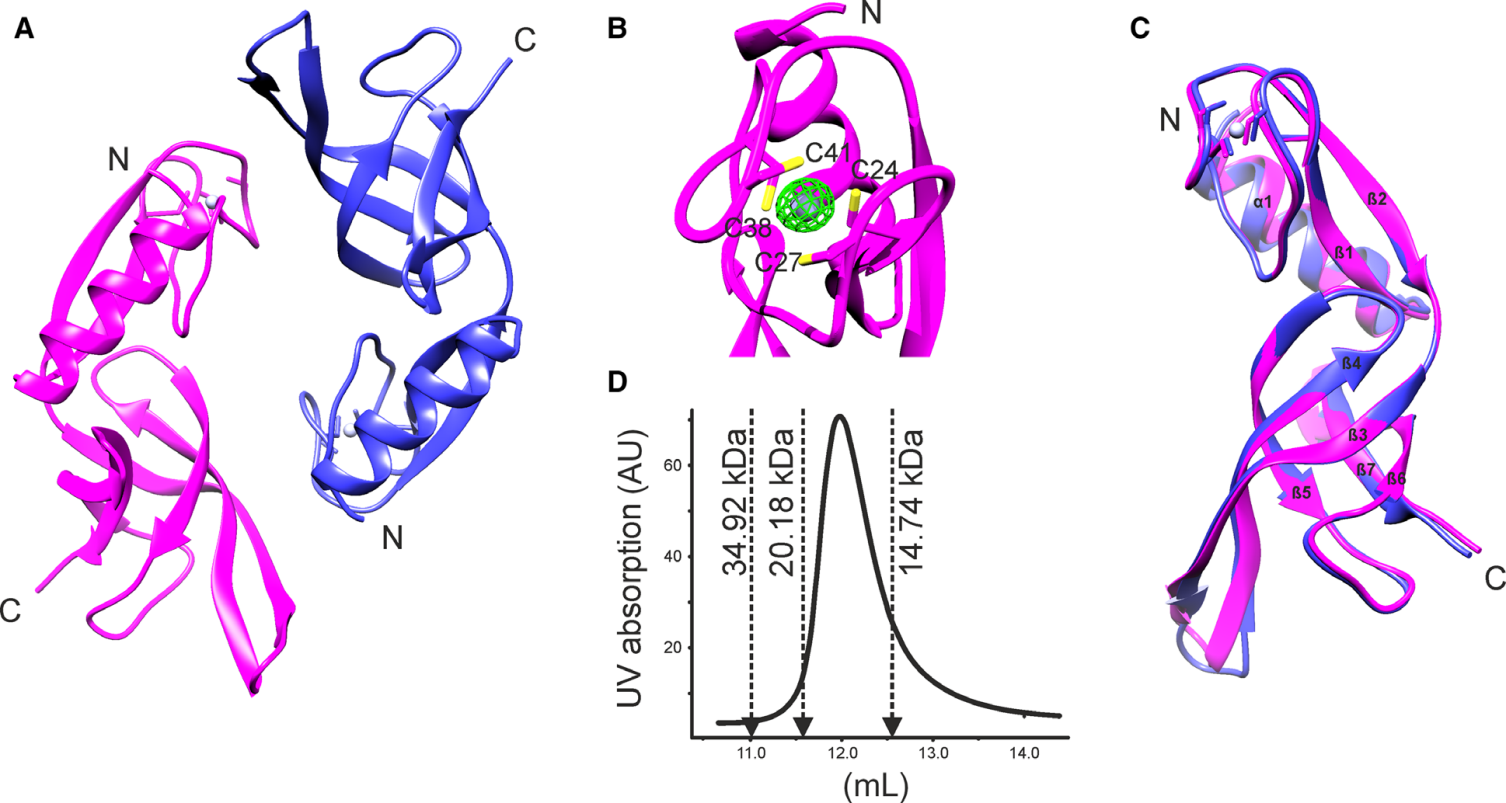
Structure of Trf. (A) Cartoon representation of the X‐ray structure for the Trf homodimer found in the crystal. Chain A is shown in magenta, chain B in medium blue, and the bound zinc ion as a blue/gravy sphere. (B) Close‐up of the zinc coordination site of Trf. The zinc‐coordinating cysteine side chains are labeled. The electron density difference map (F_o_–F_c_) for the zinc ion is shown in green at the 3σ level. (C) Superimposition of the two monomers of the crystallographic Trf dimer. Secondary structure elements are named and numbered. (D) Analytical gel filtration profile of Trf which shows that Trf is monomeric in solution. Arrows indicate the elution volumes of marker proteins in the same buffer (PhFap7: 20.18 kDa; PhS11: 14.74 kDa; PhFap7/PhS11 complex: 34.92 kDa).

Structural information for the 124 aa protein Trf responsible for the release of mRNAs bound to aIF2γ during the outgrowth phase in *S. solfataricus* was so far not available 5. Here, we report the crystal structure of Trf determined from a cuboid needle‐like crystal diffracting to a resolution of 1.65 Å (Table 1). Previously, a sequence alignment suggested a N‐terminal rubredoxin‐like domain followed by a C‐terminal OB‐fold domain 5. The X‐ray structure (Fig. 1A) confirmed that Trf contains two domains, an N‐terminal rubredoxin‐like domain (aa: 19–51) and a C‐terminal OB‐fold‐like domain (aa: 54–117). Additionally, a short α‐helix (α1, aa 3–15) precedes the rubredoxin‐like domain which is composed of two antiparallel β‐strands (β1, aa 19–24; β2, aa 46–51) surrounding a loop region (aa 25–45), which complexes a zinc ion. The zinc ion is coordinated by four cysteine residues (aa 24, 27, 38, and 41) of the loop region (Fig. 1B). The β‐barrel of the OB‐fold‐like domain is formed by five antiparallel β‐strands (β3–β7, aa 54–65; 69–78; 83–88; 99–107; and 110–117) as is typical for this type of domain 23.

**Table 1:** Data collection and refinement statistics. Statistics for the highest resolution shell are shown in parentheses.

Wavelength	1.0
Resolution range	37.44–1.65 (1.709–1.65)
Space group	P 21 21 21
Unit cell	40.291 58.493 101.366 90 90 90
Total reflections	398 915 (34 092)
Unique reflections	29 528 (2817)
Multiplicity	13.5 (12.1)
Completeness (%)	99.82 (98.26)
Mean *I*/sigma (*I*)	13.32 (1.78)
Wilson *B*‐factor	17.80
*R*‐merge	0.1447 (1.238)
*R*‐meas	0.1504 (1.292)
*R*‐pim	0.04051 (0.3628)
CC1/2	0.998 (0.662)
CC*	1.0 (0.893)
Reflections used in refinement	29 528 (2817)
Reflections used for *R*‐free	1476 (140)
*R*‐work	0.1915 (0.2793)
*R*‐free	0.2070 (0.3074)
CC (work)	0.959 (0.823)
CC (free)	0.966 (0.814)
Number of nonhydrogen atoms	2219
Macromolecules	1964
Ligands	2
Solvent	253
Protein residues	237
RMS (bonds)	0.012
RMS (angles)	1.18
Ramachandran favored (%)	99.14
Ramachandran allowed (%)	0.86
Ramachandran outliers (%)	0.00
Rotamer outliers (%)	0.00
Clashscore	3.57
Average *B*‐factor	25.74
Macromolecules	24.79
Ligands	13.77
Solvent	33.17

The asymmetric unit contains two molecules (chains A and B; Fig. 1A). For both molecules, the refined model begins with residue 1 and terminates with residue 119. The C‐terminal residues His120, Leu121, His122, Asn123, and Phe124 could not be resolved due to insufficient electron density suggestive of a flexible C terminus. Overall, the structure contains the two protein chains A and B, two zinc ions, and 257 water molecules. Both monomers of the unit cell are virtually identical with an overall RMSD for Cα atoms 1–119 of 0.739 Å (Fig. 1C). However, the dimer interface corresponds to a buried solvent‐accessible surface area of only 789 Å^2^ as determined with PDBePISA. This argues against the formation of a stable Trf dimer in solution 24. The retention volume of Trf on an analytical gel filtration column suggests that Trf in solution is indeed a monomeric protein (Fig. 1D).

**Figure 2:**
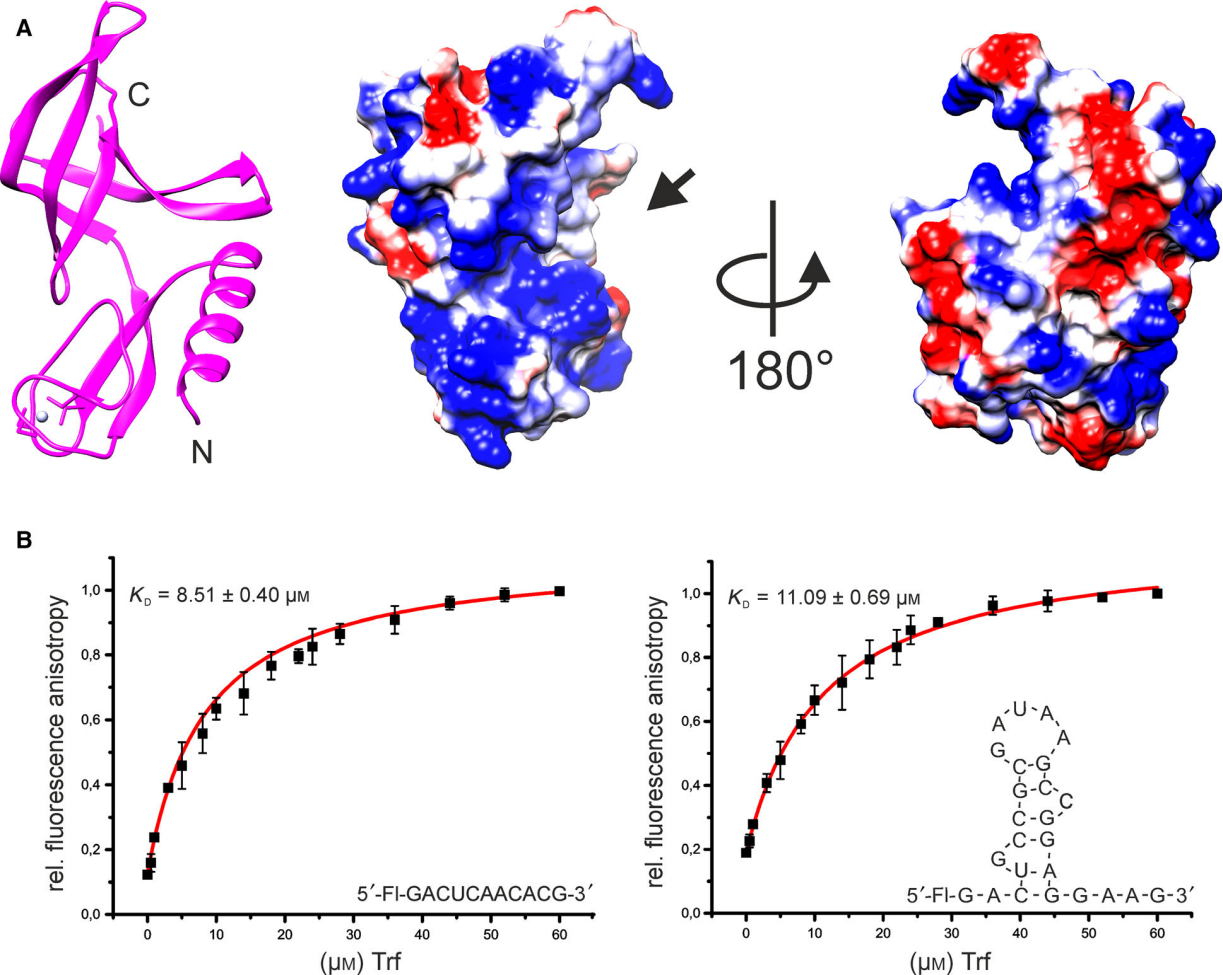
Electrostatic surface potential and RNA‐binding capability of Trf (A) Electrostatic surface potential of Trf. Blue colors correspond to positively charged areas, red colors to negatively charged areas, and white to areas with neutral electrostatic potential. For orientation, a cartoon representation of the structure of the Trf monomer in the same orientation is shown on the left. A positively charged putative RNA binding cleft is indicated by an arrow. (B) Change in fluorescence anisotropy upon titration of Trf to fluorescein‐labeled single‐stranded and hairpin RNAs. RNA sequences, secondary structures, and the resulting *K*_D_ values are depicted in the corresponding figure panels. Titration experiments were performed in triplicate. Each data point represents the average value with error bars indicating the standard deviation.

An electrostatic surface representation of Trf shows a positively charged surface for β‐strands β3, β4, and β5 which correspond to the first three β‐strands of the OB‐fold domain as well as for a part of the zinc ribbon motif (Fig. 2A). These two positively charged surface areas are located close to each other and might therefore represent a possible interaction platform for nucleic acids. This would correspond to the RNA‐binding site in many other OB‐fold proteins where often β‐strands 2 and 3 sometimes ß‐strand 1 represent the recognition and binding surface for their nucleic acid ligands 23, 25. Two other prominent determinants for RNA binding in OB‐folds are the two loops connecting β‐strands β1 and β2 (L_12_) as well as β4 and β5 (L_45_). In agreement with an RNA binding function for Trf, these loops contain a significant number of basic residues (R65 and K66 in L_12_ and K105, K110, and K111 in L_45_) augmented by aromatic residues (Y64 and Y70 in L_12_ as well as Y112 in β4) that could contribute to RNA binding by providing base stacking interactions. Furthermore, many OB‐fold proteins feature a solvent‐exposed hydrophobic residue at the center of strand 3 which is often involved in stacking interactions with nucleic acid bases 23, 25. This residue is also present in Trf as Y85.

However, in previous experiments using electrophoretic mobility shift assays (EMSA) no evidence for direct RNA binding by Trf was found 5. We tested the binding of Trf to a single‐stranded RNA and an RNA that contains a hairpin loop next to single‐stranded segments which were 5′‐fluorescein‐labeled with fluorescence anisotropy measurements. Fluorescence anisotropy measurements are sensitive to binding events over a broader *K*_D_ range compared to EMSA experiments. In titration experiments, we found that Trf bound these RNAs with *K*_D_ values in the low micromolar range (Fig. 2B). Thus, Trf apparently has a genuine RNA‐binding capacity in agreement with its proposed function. However, the additional presence of a structured hairpin loop element in one of the RNAs did not lead to an increased affinity of Trf for this RNA.

**Figure 3:**
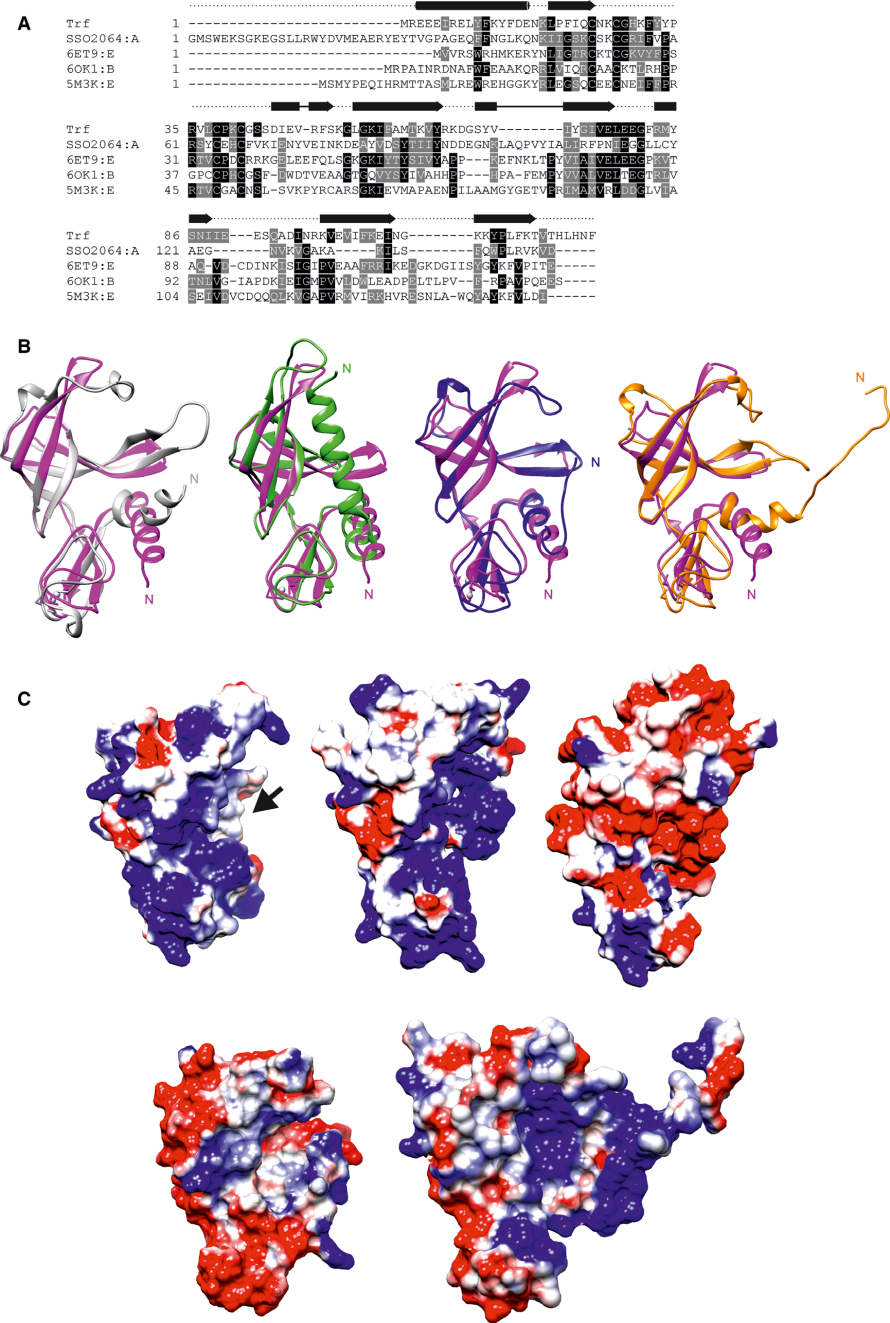
Sequence comparison and structural similarities between Trf and the other two structurally characterized DUF35 domain‐containing proteins. (A) Sequence alignment of Trf, http://www.rcsb.org/pdb/search/structidSearch.do?structureId=3irb, http://www.rcsb.org/pdb/search/structidSearch.do?structureId=6et9: chain E, http://www.rcsb.org/pdb/search/structidSearch.do?structureId=6ok1: chain B, and http://www.rcsb.org/pdb/search/structidSearch.do?structureId=5m3k: chain B. Conservation is shown in black (identical residues) and gray (similar residues). The secondary structure of Trf is indicated above the alignment by arrows and cylinders for β‐strands and α‐helices, respectively. (B) Superimpositions of http://www.rcsb.org/pdb/search/structidSearch.do?structureId=6et9: chain E (left, gray), http://www.rcsb.org/pdb/search/structidSearch.do?structureId=3irb (second from left, forest green), http://www.rcsb.org/pdb/search/structidSearch.do?structureId=6ok1: chain B (second from right, blue), and http://www.rcsb.org/pdb/search/structidSearch.do?structureId=5m3k: chain B (right, orange) onto Trf (magenta). (C) Comparison of the electrostatic surface characteristics of Trf (upper row, left), http://www.rcsb.org/pdb/search/structidSearch.do?structureId=6et9 chain E (upper row, middle), http://www.rcsb.org/pdb/search/structidSearch.do?structureId=3irb (upper row, right), http://www.rcsb.org/pdb/search/structidSearch.do?structureId=6ok1: chain B (lower row, left), and http://www.rcsb.org/pdb/search/structidSearch.do?structureId=5m3k: chain B (lower row, right). An arrow indicates the position of the positively charged putative RNA binding cleft of Trf.

To find structurally similar proteins in the PDB, we used the DALI server 20 as well as PDBeFold 21. Trf has structural homology to four other published structures (PDB http://www.rcsb.org/pdb/search/structidSearch.do?structureId=3irb, http://www.rcsb.org/pdb/search/structidSearch.do?structureId=6et9, http://www.rcsb.org/pdb/search/structidSearch.do?structureId=6ok1, and http://www.rcsb.org/pdb/search/structidSearch.do?structureId=5m3k, Fig. 3A). All four proteins also belong to the DUF35 family 7, 8, 9, 10 but apparently have no RNA‐related functions. The first example—a protein termed Sso2064—also occurs in *S. solfataricus* 7. Functionally, Sso2064 is linked to acetyl‐CoA binding related to functions in the biosynthesis of lipids and polyketide antibiotics based on a genome context analysis 7. Sso2064 also crystallized as a homodimer similar to our observations for Trf. The superimposition of Trf with Sso2064 shows a similar overall fold with a Cα RMSD of 2.3 Å for 108 aligned residues and a DALI *Z*‐score of 10.4. The second DUF35 protein was recently identified as a scaffolding subunit in the acetoacetyl‐CoA thiolase/high‐mobility group‐CoA synthase complex of *Methanothermococcus thermolithotrophicus* (pdb entry http://www.rcsb.org/pdb/search/structidSearch.do?structureId=6et9 chain E) which is part of the mevalonate pathway 8. The DUF35 subunit (chain E) in pdb entry http://www.rcsb.org/pdb/search/structidSearch.do?structureId=6et9 aligns to Trf with a Cα RMSD of 2.2 Å for 107 residues with a DALI *Z*‐score of 12.7. Another example for a DUF35 protein acting as a scaffolding subunit in a CoA‐dependent enzyme complex was found in the structure of a steroid side‐chain‐cleaving aldolase 9 from the bacterium *Thermomonospora curvata* (pdb entry http://www.rcsb.org/pdb/search/structidSearch.do?structureId=6ok1, chain B). The structure of this protein aligns to Trf with a Cα RMSD of 2.4 Å for 116 aligned residues (DALI *Z*‐score 15.4). DUF35 protein family members can also occur as a scaffolding subunit in CoA‐independent enzymes 10 as illustrated by the structure of a Friedel–Crafts acylase complex from the bacterium *Pseudomonas protegens* (pdb entry http://www.rcsb.org/pdb/search/structidSearch.do?structureId=5m3k, chain B). However, it should be noted that this enzyme complex is apparently structurally and evolutionarily related to CoA‐dependent enzymes 10. The DUF35 subunit of this complex aligns to Trf with a Cα RMSD of 2.5 Å for 110 aligned residues (DALI *Z*‐score 12.0). All DUF35 proteins structurally characterized so far bind a zinc ion in equivalent positions. In those cases where the DUF35 homolog was crystallized as a scaffolding protein in the context of larger hetero‐oligomeric assemblies (http://www.rcsb.org/pdb/search/structidSearch.do?structureId=6et9, http://www.rcsb.org/pdb/search/structidSearch.do?structureId=6ok1, and http://www.rcsb.org/pdb/search/structidSearch.do?structureId=5m3k) which are either dimers of heterodimers or dimers of heterotrimers, the DUF35 subunits are always involved in extensive interactions with the other subunits of the complex and never stabilize these complexes by homotopic interactions with each other.

In comparison with Sso2064, all other structurally characterized DUF35 family members including Trf lack an additional long N‐terminal α‐Helix (Fig. 3B). Despite the high degree of structural similarity between all proteins, the level of sequence identity between Trf and all other structurally characterized DUF35 proteins is rather low (22% for Sso2509, 24% for chain E of http://www.rcsb.org/pdb/search/structidSearch.do?structureId=6et9, 22% for chain B of http://www.rcsb.org/pdb/search/structidSearch.do?structureId=6ok1, and 19% for chain B of http://www.rcsb.org/pdb/search/structidSearch.do?structureId=5m3k). A sequence alignment of the proteins is shown in Fig. 3A. Importantly, the electrostatic surface potentials of the proteins differ considerably. The N‐terminal helical parts of Trf as well as of chain E of http://www.rcsb.org/pdb/search/structidSearch.do?structureId=6et9 show a positive electrostatic surface potential, whereas the N‐terminal helices of Sso2064 feature a negative surface potential due to the presence of an additional long N‐terminal α‐helix in this protein (Fig. 3C).

Furthermore, the parts of both domains forming the putative ‘binding cleft’ display a positive surface potential in Trf as well as in chain E of http://www.rcsb.org/pdb/search/structidSearch.do?structureId=6et9 and chain B of http://www.rcsb.org/pdb/search/structidSearch.do?structureId=5m3k, while Sso2064 and chain B of http://www.rcsb.org/pdb/search/structidSearch.do?structureId=6ok1 show a negative surface potential in this area (Fig. 3C). Thus, in regard to its overall structure and its surface properties Trf is most similar to chain E of http://www.rcsb.org/pdb/search/structidSearch.do?structureId=6et9. Interestingly, in chain E of http://www.rcsb.org/pdb/search/structidSearch.do?structureId=6et9 the positively charged ‘binding cleft’ between the two domains interacts with the thiolase domain of the enzyme complex. For Trf, this binding cleft could be either the interaction surface for aIF2γ or for its target mRNAs. However, since *S. solfataricus* aIF2γ features only a rather limited area of negative surface potential compared to the thiolase domain of *M. thermolithotrophicus*, a potential mRNA binding surface seems to be more likely. In analogy to the function of chain E of http://www.rcsb.org/pdb/search/structidSearch.do?structureId=6et9, Trf might function in order to establish a transient scaffolding state between Trf, aIF2γ, and the bound mRNA to promote the release of bound mRNAs from aIF2γ.

Overall, our results demonstrate that while sequentially dissimilar DUF35 proteins fold into highly similar structures, they differ considerably in their surface features and can therefore be recruited into a potentially wide variety of biological pathways with general scaffolding and binding functions.

## Conflict of interest

The authors declare no conflict of interest.

## Author contributions

MK overexpressed, purified, and crystallized protein; analyzed data; and wrote the manuscript. JPW provided protocols; assisted in purification, crystallization, and data analysis; and discussed the manuscript. BM provided reagents and protocols. UB provided reagents and protocols, conceptualized the project, analyzed data, and discussed the manuscript. DP collected, analyzed diffraction data, and discussed the manuscript. JW conceptualized the project, analyzed data, and wrote the manuscript.

## Data Availability

The structure of *S. solfataricus* Trf has been deposited in the PDB under accession code http://www.rcsb.org/pdb/search/structidSearch.do?structureId=6HTJ.
